# Systemic immune-inflammation index as a novel predictor of major adverse cardiovascular events in patients undergoing percutaneous coronary intervention: a meta-analysis of cohort studies

**DOI:** 10.1186/s12872-024-03849-4

**Published:** 2024-04-01

**Authors:** Chunyu Zhang, Minghao Li, Lin Liu, Li Deng, Xie Yulei, Yi Zhong, Bin Liao, Lu Yu, Jian Feng

**Affiliations:** 1 Department of Cardiology, The Affiliated Hospital of Southwest Medical University, Stem Cell Immunity and Regeneration Key Laboratory of Luzhou, Luzhou, Sichuan China; 2 Department of Rheumatology, The Affifiliated Hospital of Southwest Medical University, Luzhou, Sichuan China; 3 School of Rehabilitation, Capital Medical University, Beijing, China; 4 Department of Rehabilitation Medicine, Affiliated Hospital of North Sichuan Medical College, Sichuan, China; 5 Department of Cardiovascular Surgey, The Afilated Hospital of Southwest Medical University, Metabolic Vascular Diseases Key Laboratory of Sichuan Province, Luzhou, Sichuan China; 6 Department of Respiratory Medicine, Sichuan Academy of Medical Sciences, Sichuan Provincial People’s Hospital, Chengdu, China

**Keywords:** Percutaneous coronary intervention, Systemic immune-inflammation index, Prognosis, Meta-­analysis

## Abstract

**Background:**

The Systemic Immune-Inflammation Index (SII), a novel marker of inflammation based on neutrophil, platelet, and lymphocyte counts, has demonstrated potential prognostic value in patients undergoing percutaneous coronary intervention (PCI). Our aim was to assess the correlation between the SII and major adverse cardiovascular events following percutaneous coronary intervention.

**Methods:**

We searched PubMed, Web of Science, Embase, and The Cochrane Library from inception to November 20, 2023, for cohort studies investigating the association between SII and the occurrence of MACEs after PCI. Statistical analysis was performed using Revman 5.3, with risk ratios (RRs) and 95% confidence intervals (CIs) as relevant parameters.

**Results:**

In our analysis, we incorporated a total of 8 studies involving 11,117 participants. Our findings revealed that a high SII is independently linked to a increased risk of MACEs in PCI patients (RR: 2.08,95%CI: 1.87–2.32, *I*^*2*^ = 42%, *p* < 0.00001). Additionally, we demonstrated the prognostic value of SII in all-cause mortality, heart failure, and non-fatal myocardial infarction.

**Conclusions:**

Elevated SII may serve as a potential predictor for subsequent occurrence of MACEs in patients undergoing PCI.

**Trial registration:**

Our protocol was registered in PROSPERO (registration number: CRD42024499676).

## Introduction

Coronary artery atherosclerotic disease is recognized as a primary contributor to illnesses and mortality in the elderly population [1], with a mortality rate constituting around 30% of total deaths [2]. Among them, acute coronary syndrome(ACS) is regarded as the primary subtype of the disease. With the rising burden of ischemic heart disease, percutaneous coronary intervention (PCI) has emerged as a primary therapeutic approach for acute coronary syndrome [3]. Despite the continuous breakthroughs in modern PCI technology, drug-eluting stents, and antiplatelet therapy, many patients still face various cardiovascular complications after undergoing PCI treatment [4, 5] such as cardiogenic shock [6], all-cause mortality [7], non-fatal myocardial infarction [8], non-fatal stroke [9] and repeat revascularization [10], among other adverse cardiovascular events. Such a scenario has the potential to significantly jeopardize the future survival and quality of life of patients. Hence, it is of paramount importance to identify patients actively undergoing PCI treatment, yet still at a heightened risk of adverse cardiovascular events.

Atherosclerosis represents a chronic inflammatory vascular disease with systemic implications [11, 12]. In recent years, Evidence from clinical practice supports the role of the neutrophil-to-lymphocyte ratio (NLR) and platelet-to-lymphocyte ratio (PLR) as predictors of prognosis in cardiovascular disease. Hu et al. introduced the Systemic Immune-Inflammation Index (SII) in 2014, a comprehensive inflammatory assessment tool calculated as SII = (neutrophil × platelet) / lymphocyte [13]. This index determines the immune and inflammatory status by comprehensively evaluating neutrophil, platelet, and lymphocyte counts obtained from routine complete blood cell analysis. Currently, SII has been confirmed as an independent prognostic factor for various cancers [13–15], and research has found that SII also has a good predictive role in cardiovascular diseases [16]. Further studies indicate that, in predicting cardiovascular disease outcomes, SII may have better prognostic value compared to NLR and PLR [17]. Yang et al.'s research revealed an independent association between the SII and the occurrence of major adverse cardiovascular events in patients with Coronary Artery Disease (CAD) following coronary artery intervention [16]. Faysal Saylik et al. found that SII can effectively predict the occurrence of major adverse cardiovascular events (MACEs) in patients with ST-segment elevation myocardial infarction (STEMI) after undergoing PCI treatment [18]. However, there is currently a lack of comprehensive systematic analysis regarding the relationship between SII and MACEs after PCI treatment. Therefore, we conducted a meta-analysis to thoroughly investigate the relationship between SII and MACEs after PCI treatment by integrating current research findings, aiming to provide guidance for future research and clinical practice.

## Methods

### Search strategy

Adhering to the Preferred Reporting Items for Systematic Reviews and Meta-Analyses (PRISMA) guidelines, our systematic review and meta-analysis were conducted [19]. Our protocol was registered in PROSPERO (registration number: CRD42024499676). Up to November 20, 2023, articles from four English databases (PubMed, Embase, Web of Science, and The Cochrane Library) were retrieved, with language restrictions. using keywords including "systemic immune-inflammation index", "SII", "coronary artery disease", "myocardial infarction", "acute coronary syndrome", "percutaneous coronary intervention", "Percutaneous transluminal coronary angioplasty", "STEMI", "NSTEMI", "PCI", "PTCA", "AMI", "ACS" and "major adverse cardiovascular and cerebrovascular events". Furthermore, manual searches were conducted, involving the examination of reference lists from prior systematic reviews and meta-analyses, to pinpoint relevant articles for in-depth analysis.

### Study selection

Independently, two investigators (ZCY and LMH) evaluated the methodological quality of the included studies. If discrepancies were identified, we recorded and negotiated with the third investigators (LL) to resolve the differences. The inclusion criteria for this study were: (1) Study type: retrospective or prospective cohort studies; (2) Study population: patients undergoing PCI; (3) The primary outcome, defined as a composite of cardiovascular death, non-fatal myocardial infarction, non-fatal stroke, repeat revascularization, and heart failure, is MACEs; (4) Secondary outcome measures encompass all-cause mortality, myocardial infarction, non-fatal stroke, heart failure, and repeat revascularization.

Exclusion: (1) Excluded from the analysis were cross-sectional studies, reviews, preclinical investigations, and studies not aligned with the meta-analysis objectives; (2) Animal experiments, conference papers, case reports, and duplicate publications were excluded; (3) Studies that did not provide outcome indicators for MACEs after SII grouping were excluded.

### Data extraction and quality assessment

Initially, duplicate articles were excluded, and the remaining retrieved papers underwent independent screening by two researchers. Through the review of titles and abstracts and the application of consistent inclusion and exclusion criteria, articles meeting the criteria underwent a meticulous screening process. Following a thorough full-text analysis, articles with insufficient information in their abstracts were scrutinized. Any discrepancies were resolved through discussions or negotiations, often requiring the input of a third researcher.

The collected data encompassed: (1) Author's name, publication year, and country of origin; (2) Study design characteristics; (3) Patient attributes, encompassing diagnosis, sample size, age, and gender distribution; (4) SII index analysis approach; (5) Duration of follow-up; (6) Outcomes of adverse events. The quality assessment employed the Newcastle–Ottawa Scale (NOS), evaluating cohort study quality based on three criteria: group selection, group comparability, and outcome determination. Scores on the NOS range from 1 to 9 stars. Those with a NOS score of 6 were considered to be of high quality [20].

### Statistical analysis

In the statistical analysis, the risk ratio (RR) and its corresponding 95% confidence interval (CI) served as the standard measurements to assess the correlation between SII and the risk of adverse events in PCI patients. For studies analyzing SII as a categorical variable, we extracted data on major adverse cardiovascular events from the highest and lowest SII groups for statistical analysis. To demonstrate the potential independent association between SII and MACEs occurrence rate in PCI patients, we only extracted and combined RR data from the most extensively adjusted multivariate analysis models. To assess heterogeneity among the included cohort studies, we utilized Cochrane's Q test and calculated the* I*^*2*^ statistic [21], Acknowledging significant heterogeneity when *I*^*2*^ > 50%, the synthesis of risk ratio data was performed using a random-effects model. This model was selected for its broader applicability in accommodating potential heterogeneity among the included studies [22]. Sensitivity analysis, systematically excluding one individual study at a time, was conducted to assess result stability [23]. Statistical significance was set at *P* < 0.05. Assessment of potential publication bias involved a visual examination of funnel plot symmetry and the application of Egger's test [24]. Analysis was performed using RevMan software (version 5.1; Cochrane Collaboration, Oxford, UK).

## Results

### Study selection and study characteristics

**Fig. 1:**
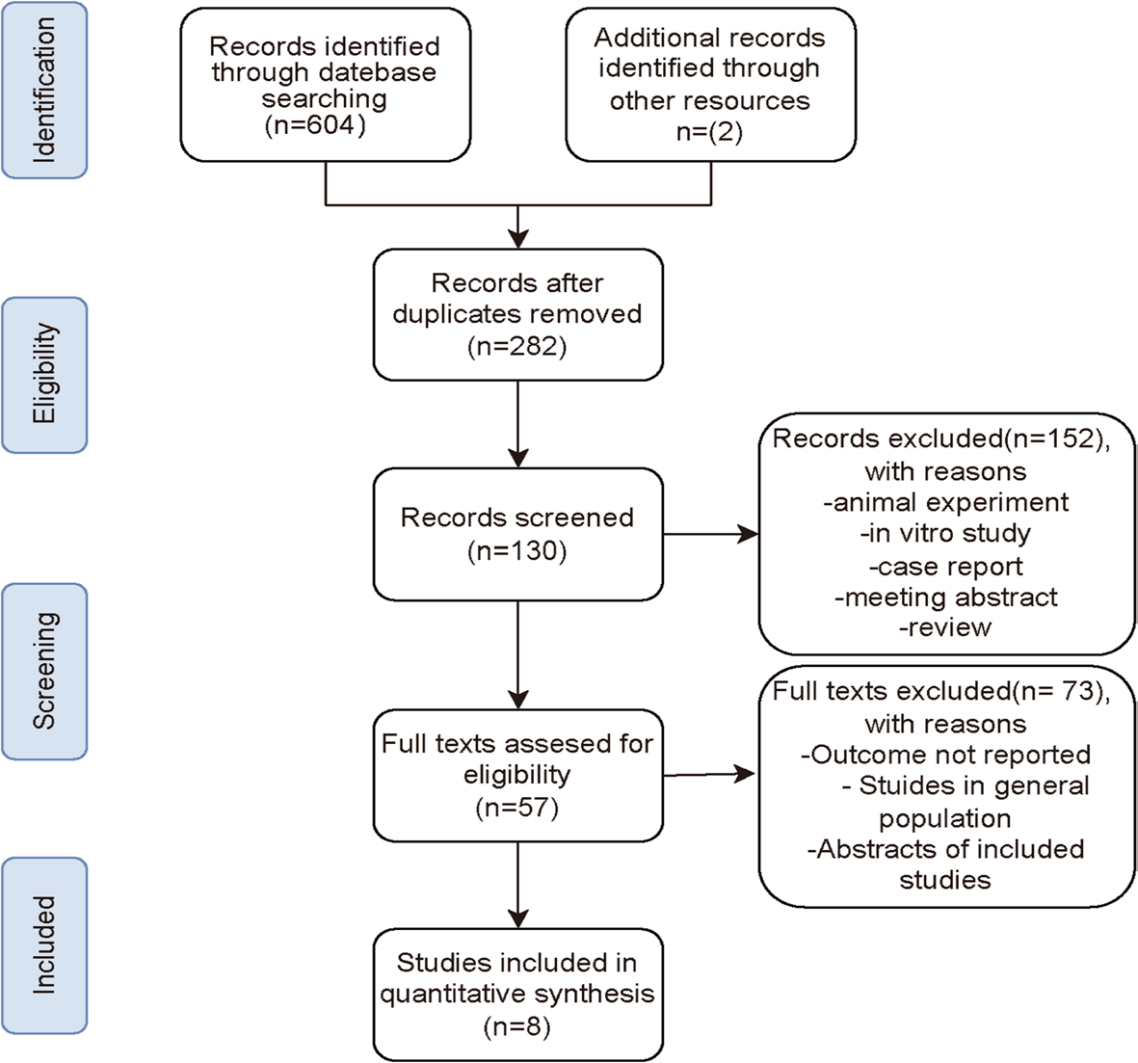
PRISMA flowchart of the study selection process

From PubMed, Embase, Web of Science, and The Cochrane database, a total of 604 records were obtained. By manual retrieval, two more articles were added, resulting in a total of 8 studies that met the eligibility criteria for analysis based on inclusion and exclusion criteria [16, 18, 25–30]. Figure 1 presents the flowchart outlining the process of study selection and the reasons for exclusion after a full-text examination. Initially, 324 duplicate publications were removed using reference management software (EndNote X7). Subsequently, 152 articles were excluded due to animal experiments, case reports, reviews, or summaries. Then, 57 publications were identified for full-text review. After further screening, 8 cohort studies, including 11,117 participants, were used for subsequent meta-analysis. The participants had an average/median age spanning from 56.93 to 75.47 years. The conducted studies were published in two regions: Turkey and China. The cutoff values for SII were determined using ROC analysis, the Youden index, tertiles, and quartiles. Table 1 offers a comprehensive summary of the characteristics of the included studies. Six studies scored between 7 and 8 on the NOS scale, Signifying a reduced bias risk. Two studies received a score of 6, primarily due to an increased bias risk resulting from insufficient comparability caused by unaddressed confounding factors (Table 2).

**Table 1:** Characteristics of studies investigating the relationship between systemic immune-inflammation index (SII) and PCI

Study	Country	Year	Involved centers	Design	SII analysis	SII cut-off value	Age(mean + SD)	Sample size	HTN%	DM%	HPL%	Follow-up duration (days)	Outcome
Ya-Ling Yang	china	2020	single	RC	High SII: Low SII	694.3	69.2 ± 12.9	5602	87	40	NA	1638	①③④⑤⑥
Demet Ozkaramanli Gur	Turkey	2021	single	RC	T3:T1	NA	64.15 ± 11,56	206	36	40	43	365	①
Sanling Shi	china	2022	single	RC	High SII: Low SII	1159.84	71.9 + 10.7	744	77	40	49	912.5	①②③④⑤⑥
Gokhan Demirci	Turkey	2023	single	RC	High SII: Low SII	548	63.6 ± 9.1	303	78	44	56	10,095	①
Faysal Saylik	Turkey	2021	single	RC	High SII: Low SII	951.7	60.2 ± 9.74	843	33	24	39	1026	①②③④⑥
Lütfi Öcal	Turkey	2021	single	RC	High SII: Low SII	1781	56.93 ± 11.53	1660	42	22	27	999	①②④⑥
Wenjun Fan	china	2021	single	PC	High SII: Low SII	628.60	NA	1553	59	25	22	1142	①
Xing Wei	china	2023	single	RC	T3:T1	1,085.55	62.54 ± 13.76	310	58	31	NA	NA	①

①MACEs, ②all-cause mortality, ③non-fatal stroke, ④non-fatal MI, ⑤heart failure, ⑥repeat revascularization

*Abbreviations: SII* systemic immune-inflammation index, *RC* Retrospective cohort, *ACS* acute coronary syndrome, *DM* diabetes, *HTN* hypertension, *HPL* hyperlipidemia, *PCI* percutaneous coronary intervention, *STEMI* ST­segment elevation myocardial infarction, *NSTEMI* Non-ST-segment elevation myocardial infarction

**Table 2:** Details of study quality evaluation via the Newcastle–Ottawa Scale

Studies	Selection				Comparability	Outcome			Total
Author/Year	Representativeness of the exposed cohort	Selection of the non-exposed cohort	Ascertainment of exposure	Outcome of interest was not present at start of study		Assessment of outcome	Long enough follow-up for outcomes to occur	Adequacy of follow up of cohorts	
Ya-Ling Yang 2020 [16]	1	1	1	0	1	0	0	1	6
Demet Ozkaramanli Gur 2021	1	1	1	1	0	0	1	1	7
Sanling Shi 2022	1	1	0	1	1	1	1	1	8
Gokhan Demirci 2023 [27]	1	1	0	1	1	1	1	1	8
Faysal Saylik 2021	1	0	1	0	1	0	1	1	6
Lütfi Öcal 2021	1	1	0	1	1	1	1	1	8
Wenjun Fan 2021 [29]	1	0	1	0	1	1	1	1	7
Xing Wei 2023 [30]	1	1	1	1	1	0	1	1	8

### Major adverse cardiovascular events

**Fig. 2:**
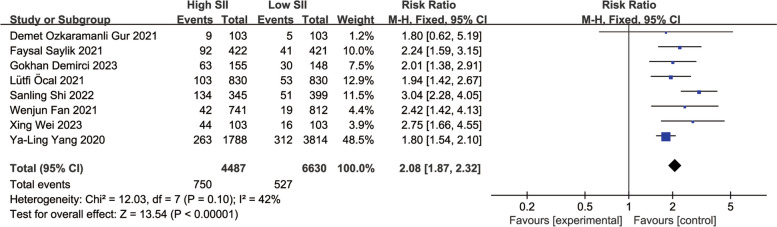
Forest plot for the associations between SII and MACEs in patients with PCI

A total of 8 observational studies were included, comprehensively analyzing data from 11,117 participants to determine the relationship between SII and MACEs during follow-up periods ranging from 1 year to 3.1 years. Compared to the lowest SII group, the highest SII group had a significantly higher risk of MACEs after PCI. The summary results of the fixed-effect model showed that the risk of MACEs after PCI in the highest SII group was 2.08 times that of the lowest group (RR: 2.08, 95% CI: 1.87–2.32, I2 = 42%, *p* < 0.00001) (Fig. 2).

### Single adverse event

**Fig. 3:**
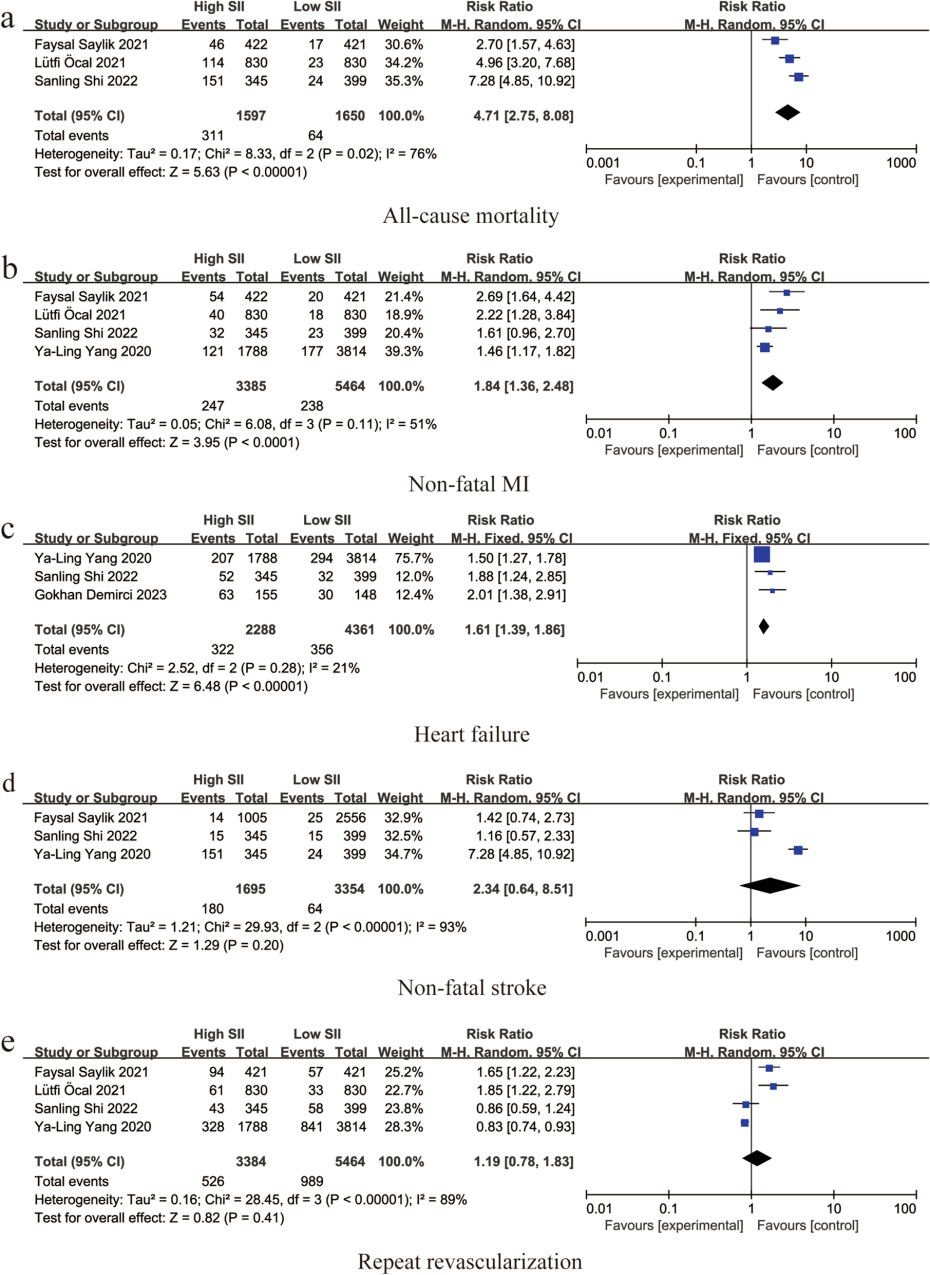
Forest plot for the associations between SII and different cardiovascular adverse events in patients with PCI: **a** Forest plot for the associations between SII and all-cause mortality in patients with PCI: **b** Forest plot for the associations between SII and non-fatal MI in patients with PCI: **c** Forest plot for the associations between SII and heart failure in patients with PCI: **d** Forest plot for the associations between SII and non-fatal stroke in patients with PCI: **e** Forest plot for the associations between SII and repeat revascularization in patients with PCI

Three studies reported an association between SII and all-cause mortality (RR: 4.71, 95% CI: 2.75–8.08, *I*^*2*^ = 76%, *p* < 0.00001) (Fig. 3a). Four studies reported an association between SII and non-fatal myocardial infarction (RR: 1.84, 95% CI: 1.36–2.48, *I*^*2*^ = 51%, *p* < 0.0001) (Fig. 3b). Three studies reported an association between SII and heart failure (RR: 1.61, 95% CI: 1.39–1.86, *I*^*2*^ = 21%, *p* < 0.00001) (Fig. 3c). An association between SII and non-fatal stroke was reported in three studies (RR: 2.34, 95% CI: 0.64–8.51, *I*^*2*^ = 93%, *p* = 0.20) (Fig. 3d). Four studies reported an association between SII and repeat revascularization (RR: 1.19, 95% CI: 0.78–1.83, *I*^*2*^ = 89%,* p* = 0.41) (Fig. 3e).

### Sensitivity analysis and publication bias

**Fig. 4:**
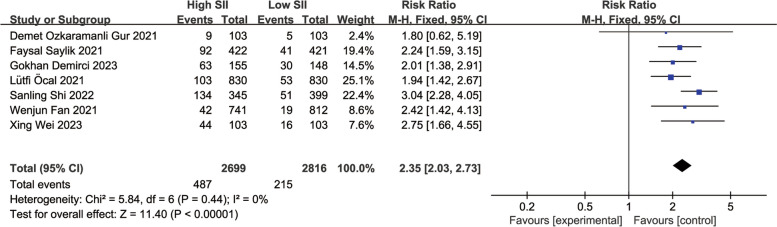
Forest plot for the associations between SII and MACEs in patients with PCI after excluded from Ya-Ling Yang 2022

Sensitivity analysis of the main outcome indicators showed that the heterogeneity mainly stemmed from the study by Ya-Ling Yang [16]. After excluding this study, the heterogeneity decreased to 0 (Fig. 4) (RR: 2.35, 95% CI: 2.03–2.73, I2 = 0%, *p* < 0.00001). Upon analyzing the included literature, we found that the study population in the study by Ya-Ling Yang had a hypertension prevalence of 87%, much higher than in the other included studies. Furthermore, the population selected in this study included stable coronary artery disease patients, which may have led to the occurrence of heterogeneity.

### Publication bias

Due to the small number of included studies (*n* < 10), this study cannot perform publication bias and subgroup analysis according to established guidelines.

## Discussion

In this meta-analysis, we included 8 cohort studies, primarily focusing on the relationship between SII and the risk of adverse cardiovascular events after undergoing PCI. The results of the study indicate that patients in the high SII group have a higher risk of experiencing MACEs after undergoing PCI compared to those in the low SII group (RR: 2.08, 95% CI: 1.87–2.32, I2 = 42%, *p* < 0.00001). Additionally, we also demonstrated the association between high SII and the occurrence of all-cause mortality, heart failure, and non-fatal myocardial infarction after undergoing PCI. The findings of this study suggest that SII can serve as an indicator for identifying high-risk populations after undergoing PCI treatment.

Our study indicates a correlation between high SII and the risk of MACEs after PCI. Although PCI is a therapeutic measure, it further exacerbates the inflammatory response in patients' bodies. Bibek et al. found that the pre-treatment inflammation level in PCI patients is closely related to short-term and long-term complications [31], and SII reflects the level of inflammation in the body to some extent. Initially, SII was used to predict tumor progression and adverse survival outcomes in different types of malignancies [32, 33]. These findings prompted researchers to further explore the role of SII in the cardiovascular field. Ma et al. conducted a large cross-sectional study involving 15,905 patients, and the results showed that higher SII values may be associated with a higher incidence of coronary heart disease [34, 35]. Dziedzic et al. found an association between SII and the incidence rate of acute coronary syndrome [36]. Liu et al. found a positive correlation between SII and the severity of coronary artery stenosis [37]. Lütfi et al.'s study also demonstrated that SII can effectively predict in-hospital and long-term mortality rates in STEMI patients [28]. The above studies may partially explain the potential association between higher SII levels in PCI patients and increased subsequent MACE risk. From a pathophysiological perspective, SII is a new indicator of systemic inflammation based on neutrophil, platelet, and lymphocyte counts. Neutrophils are the most abundant subtype of white blood cells in the circulation. Neutrophils enhance monocyte adhesion and transform into atherosclerotic plaques, releasing myeloperoxidase, NADPH oxidase, lipoxygenase, and neutrophil extracellular traps (NETs), thereby promoting endothelial dysfunction and vascular wall degeneration [38, 39]. Higher platelet counts reflect destructive inflammatory processes in the body [40], and activated platelets promote thrombosis by secreting thromboxane A2 and adenosine diphosphate [41]. Multiple studies have confirmed that increased platelet activity in PCI patients is associated with an increased risk of short-term and long-term MACEs [41–43]. CD4 + T lymphocytes belong to the regulatory arm of the immune system, playing a role in controlling immune responses and reducing myocardial damage in vivo [44]. Current research has confirmed that an increased NLR before PCI treatment is an independent predictor of three-year mortality rate and MACEs in patients [45]. Higher PLR has also been proven to be a powerful predictor of adverse cardiovascular events [46–48]. Compared to PLR and NLR, SII can more comprehensively and balancedly reflect human immune and inflammatory responses [49]. Erdoğan et al. found that SII is a more predictive inflammatory marker than NLR and PLR [50]. Additionally, Candemir M et al. found that compared to NLR and PLR, SII can better predict the severity of coronary artery lesions [51].

Currently, in clinical practice, Gensini score and SYNTAX score are commonly used to assess the risk of short-term and long-term adverse cardiovascular events in patients undergoing PCI [52, 53]. SII is closely related to the above two scores. Huang et al. found a positive correlation between SII and Gensini score [54]. Demet Ozkaramanli Gur et al. also confirmed a positive correlation between SII and SYNTAX [55]. Some researchers have begun to combine SII with other relevant indicators to enhance its predictive value. For example, results from Wang et al. [56] showed that combining SII with GRACE score can more accurately predict the occurrence of short-term MACEs after PCI in STEMI patients. Additionally, Zhu et al. found that high SII and high CHA2DS2-VASC score are risk factors for CI-AKI, and their combination can improve the accuracy of predicting CI-AKI in ACS patients undergoing PCI [57]. Therefore, in the future, clinicians can develop individualized diagnosis, treatment, and prevention strategies based on the SII value of patients before undergoing PCI, especially for high-risk patients.

There are still some limitations in this study. Firstly, current studies on the association between SII and PCI risk have used different SII cutoff values, so standardization of SII is needed before its widespread use. Secondly, limited by the fact that all included studies were retrospective and single-center, and the number of included studies was small, we were unable to perform publication bias tests, which may lead to inherent clinical heterogeneity. Lastly, the included studies were only conducted in China and Turkey, so caution is needed when applying the results to other regions or populations. Therefore, in the future, we hope for more randomized controlled trials with larger samples from different regions to validate the applicability of our conclusions.

## Conclusions

In conclusion, current cohort studies suggest that elevated SII may serve as a potential predictor for subsequent occurrence of MACEs in patients undergoing PCI.

## Data Availability

The data used to support the findings of this study are included within the article.

## Abbreviations

SII: Systemic Immune-Inflammation Index
ACS: Acute coronary syndrome
PCI: Percutaneous coronary intervention
NLR: Neutrophil-to-lymphocyte ratio
PLR: Platelet-to-lymphocyte ratio
CAD: Coronary Artery Disease
MACEs: Major adverse cardiovascular events
NOS: The Newcastle–Ottawa Scale
RR: Risk ratio
CI: Confidence interval
DM: Diabetes
HTN: Hypertension
HPL: Hyperlipidemia
STEMI: ST­segment elevation myocardial infarction
NSTEMI: Non-ST-segment elevation myocardial infarction
